# A picture is worth 1000 pathological learning points: Creating visual pathology teaching materials appropriate for preclerkship medical student learning

**DOI:** 10.1016/j.acpath.2026.100305

**Published:** 2026-10-17

**Authors:** Phoebe M. Hammer, Krishna L. Bharani, Aubre Gilbert, Ahmed Afifi, Josselyn Sofia Vergara Cobos, Jaclyn B. Anderson, Cindy Wang, Rebecca Kate Miller-Kuhlmann, Kelly Ernst

**Affiliations:** a Oregon Health & Science University, Department of Pathology, Portland, OR, USA; b Stanford University, Department of Pathology, Stanford, CA, USA; c The Ohio State University Wexner Medical Center, Department of Pathology, Columbus, OH, USA; d Benha University, Department of Pathology, Al-Qalyubia Governorate, Egypt; e Stanford Univeristy, Department of Neurology, Stanford, CA, USA

**Keywords:** Education, Gross, Medical students, Pathology, Histology, Teaching, Undergraduate

## Abstract

Quality teaching materials covering gross and microscopic pathology at an appropriate complexity level for preclerkship medical students are lacking. At our institution, feedback regarding the preclerkship course curriculum identified a need for clearer and more clinically relevant visual pathology teaching materials. Educators in the Pathology Department and School of Medicine collaborated to design PowerPoint slide decks emphasizing the interpretation of gross and microscopic pathology images at a level appropriate for preclerkship medical students that aligned with the course curriculum, including small-group learning sessions. Topics included endocrine, reproductive, gastrointestinal, liver, heart, lung, renal, musculoskeletal, neurology, and skin. Images demonstrating pathology were selected from digital image databases and annotated with clinical pearls and side-by-side comparison with normal examples. Five-hundred and eleven annotated PowerPoint slides were created for ten distinct systems-based modules, covering 70 disease entities. Survey questions regarding the modules were included in required course evaluations. The material was well received by two student cohorts who overall said the material was good, very good, or excellent for learning (57/66, 86%) and agreed or strongly agreed that it was at the appropriate educational level (50/70, 71%). In collaboration with specialty clinicians and pathologists, we harnessed digital gross and histology images to directly address gaps in pathology education identified by preclerkship medical students.

## Introduction

In the last two decades, most medical schools have transitioned to a systems-based education model in which formerly distinct disciplines, such as anatomy and histopathology, are now integrated into an interdisciplinary and clinical-focused curriculum.^1^^,^^2^ These reforms have resulted in shortened preclinical curricula with less time allotted to basic sciences.^3^ Despite the decreased separate time allotted to nonintegrated pathology, the subjects of histology, anatomy, and histopathology remain a key link between the basic sciences and clinical medicine and still represent a significant portion of content tested in the current US Medical Licensing Examination (USMLE), particularly in Step 1 and Step 3.^4^^,^^5^ Given these changes, educators in histology and histopathology must develop level-appropriate, efficient coursework with an emphasis on clinical relevance.^6^

Additionally, the structure of preclinical education is moving away from exclusively large-group didactic lectures toward more “learner-centered” active learning strategies such as the flipped classroom, small-group discussion, team-based exercises, and case presentations.^1^^,^^7^^,^^8^ Additionally, a number of self-guided, asynchronous resources are also becoming key learning tools for medical students. Numerous educational innovations have been developed to support pathology and histology education, including virtual microscopy platforms, interactive web-based atlases, adaptive tutorials, mobile applications, collaborative annotation tools, and commercial board review resources.^9^, ^10^, ^11^, ^12^, ^13^, ^14^, ^15^, ^16^, ^17^, ^18^ These resources have demonstrated benefits for learner engagement and knowledge acquisition; however, many are intended for USMLE preparation or postgraduate pathology education rather than integration into institution-specific preclerkship curricula. Few resources are specifically designed to teach early medical student learners how to recognize gross and microscopic pathology using carefully annotated still images with side-by-side comparison to normal tissue while directly reinforcing concurrent organ system instruction and clinical relevance.

At our institution, student feedback regarding the preclerkship Science of Medicine curriculum identified a need for concise, visual pathology teaching materials that aligned directly with concurrent organ systems teaching. Students specifically requested annotated gross and microscopic images with normal comparisons, guidance on identifying key histologic features, and clinical correlations to underscore relevance. The aims of our educational intervention were to address this gap by creating supplemental asynchronous learning modules that complemented the corresponding didactic and small-group curriculum, with a goal of helping undergraduate medical student learners develop a systematic approach to recognizing common pathologic findings and to connect normal and abnormal morphology. In addition, we evaluated learner perceptions regarding the educational resource, including its usefulness, utility, and appropriateness for preclerkship learners. The overall purpose of this study was to describe a successful interdisciplinary process to develop an early-learner pathology resource by integrating available digital pathology resources in direct response to student feedback.

## Materials and methods

The project was conducted at Stanford University and was approved by the institutional review board as exempt in accordance with ethical standards established by the institution (Protocol ID: 78315). At our institution, histopathology content is covered in our preclerkship Science of Medicine course (as part of a pathology thread) in didactic format delivered by a pathologist and in a small-group case-based laboratory session format facilitated by both a subspecialty clinician and a pathology trainee.

Course survey feedback from medical students led to the effort to improve pathology teaching materials. As part of routine institutional course evaluations, a free-text response prompt included in required course surveys from 2020 to 2022, including all organ system blocks, was as follows: “Please provide comments for course directors about what the course should ‘keep-stop-start’ and why” (Supplemental Material 1). All responses relating to pathology were recorded and, because of the limited number of free-text responses to survey questions, a descriptive thematic review was performed. These survey responses served as the needs assessment that motivated this project.

A list of essential normal histology images and pathologic diagnostic entities was selected by the clinician leaders of each system block, with input from pathologists specializing within that field. Discrete system-blocks that corresponded to the didactic curriculum included endocrine, reproductive, gastrointestinal, liver, cardiovascular, respiratory, renal, musculoskeletal, neurology, and skin. Emphasis was placed on pathologic entities most likely to appear in clinical practice and USMLE examinations, with particular consideration of topics recommended in the Pathology Competencies for medical education.^8^

Macroscopic and microscopic images showing examples of classic histomorphology were curated by authors PMH, KE, AG, CW, KLB, and JBA using our institution's archival pathology database (PowerPath or, more recently, the Epic Beaker Laboratory Information System). In a small subset of cases, characteristic gross images could not be identified, and with appropriate references, we used images collected from open-access web-based resources.^19^^,^^20^

Slide decks were created using Microsoft PowerPoint (Fig. 1, Fig. 2, Fig. 3). Normal gross images highlighting relevant anatomy were followed by images of normal histology. Subsequent slides demonstrated classic examples of abnormal gross and histologic pathologies. Detailed annotations were added to the images, pointing out specific histologic findings corresponding to “buzzwords” and written descriptions that may be seen on examinations or in pathology reports. The images were also annotated with interwoven definitions of key pathologic terms (e.g., metaplasia) and with relevant “Clinical Tie-ins.” All slides were reviewed by authors PMH and KE (board-certified pathologists). Subspecialty attending pathologists also reviewed the slides in their particular subspecialty to ensure accuracy and content quality. As medical students, authors AA and JSVC also provided critical feedback and guidance to ensure the content was appropriate for their level of education. The course directors and Assistant Dean also completed a review of the material before they were released to medical students.

**Fig. 1:**
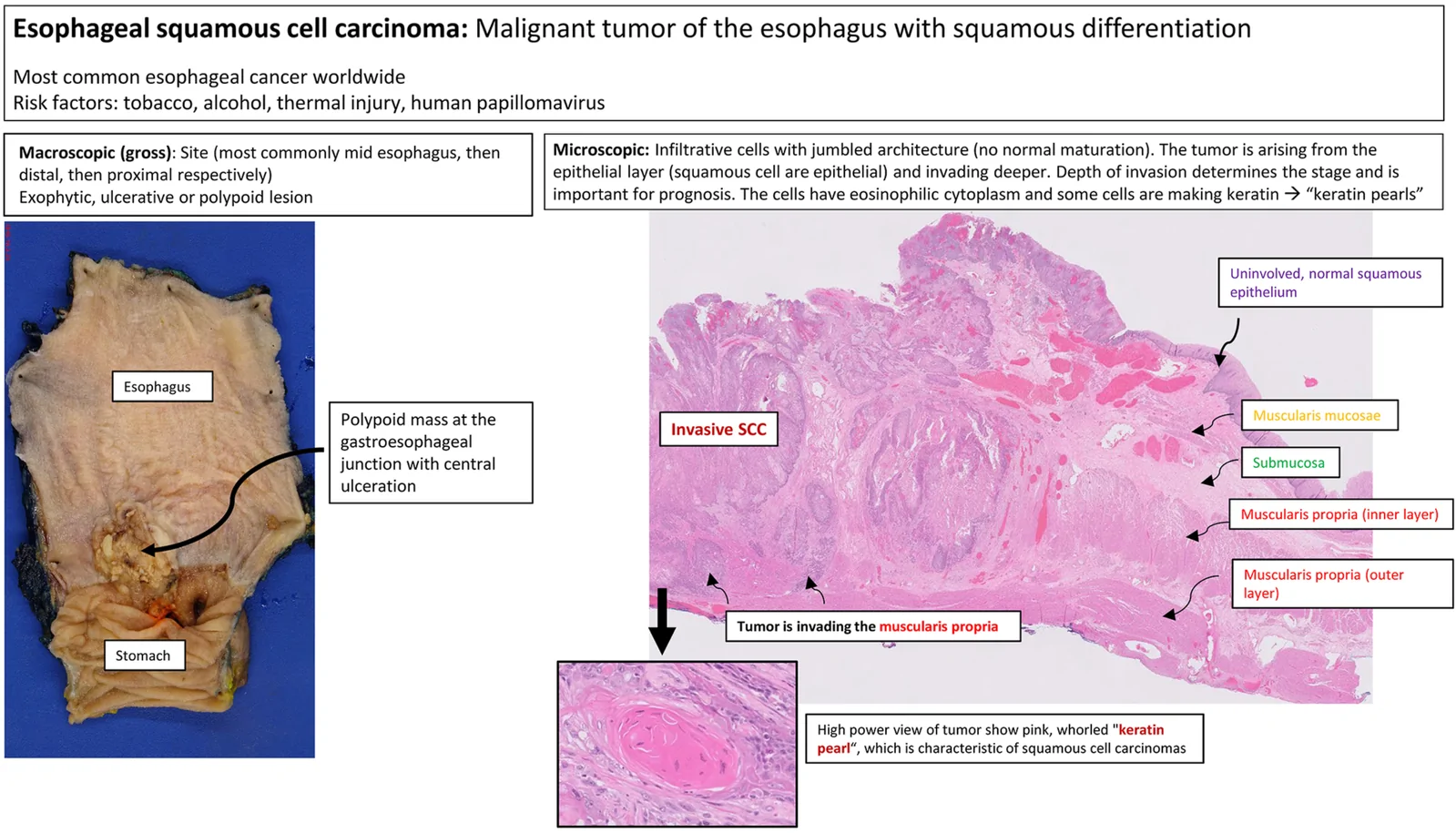
Example pathology teaching slide for preclinical medical students from the gastrointestinal block. In this slide presenting esophageal squamous cell carcinoma, important clinical features (e.g., risk factors) are noted, followed by histopathologic features. Macroscopic and microscopic findings are presented with detailed annotations and color coordinating of the layers of the esophagus in both the abnormal and normal esophagus (see Fig. 2) slides. Key histologic features for the diagnosis, in this case “keratin pearls,” are emphasized at high magnification and bold text.

**Fig. 2:**
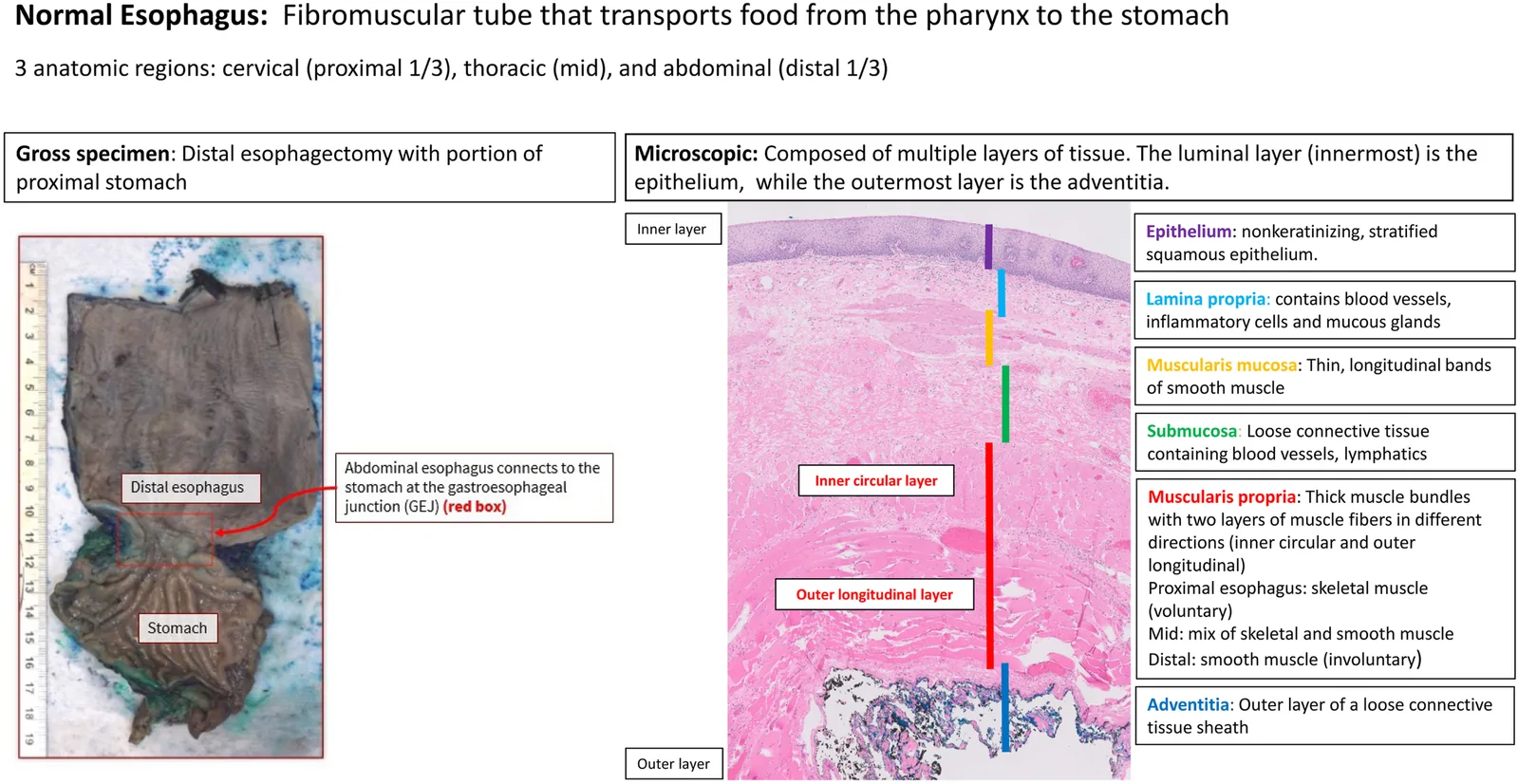
Example comparison of normal anatomy teaching slide for preclinical medical students from the gastrointestinal block. In this example, gross and microscopic features of normal esophagus are presented with detailed annotations. Annotations of the layers of the esophagus are color coordinated in the normal anatomy slide and subsequent pathology slides (Fig. 1).

**Fig. 3:**
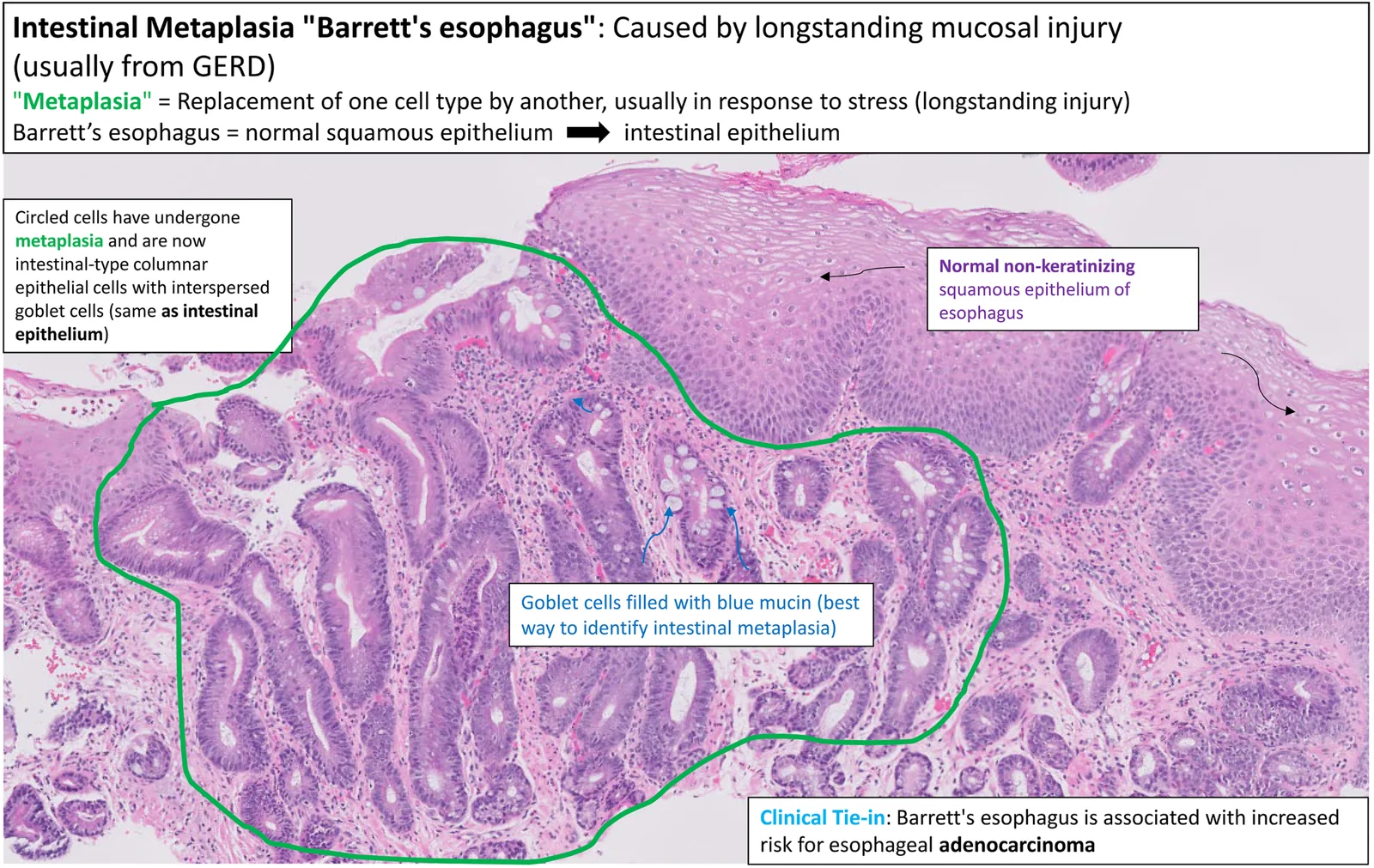
Example annotated teaching slide for preclinical medical students in the gastrointestinal block. In this example of intestinal metaplasia (“Barrett's esophagus”), a fundamental pathology concept (metaplasia) is defined, and a clinical tie-in regarding the risk of esophageal adenocarcinoma is also presented.

At the start of each system-based course, the annotated pathology slide decks became available in the student learning management system Canvas as an optional asynchronous study resource. Students were notified of the resource during each course orientation. The annotated slide decks were also used as supplemental materials during the small-group pathology laboratories, and pathologist lecturers were also encouraged to use the slides in didactic sessions as appropriate. Organ system–based blocks span a full year starting in the spring of the first year of medical school at our institution.

Following implementation of the annotated pathology slide decks, pathology-specific questions were incorporated into the institution's existing required end-of-course evaluations. A 5-point Likert scale structured question, “The annotated pathology image slide decks available on Canvas were useful for learning,” was added for first-year medical students after finishing their Spring blocks, including pulmonary, cardiovascular, and musculoskeletal blocks. Then, two 5-point Likert scale structured questions including “Please rate the utility of supplementary pathology PowerPoint PDFs i.e., “Endocrine Pathology Images”/" Renal Pathology Images" provided by Pathologists.” and “The annotated pathology PowerPoint slides were appropriate for my learning level for histopathology” plus a free-text response “Please provide feedback for the Pathology Department about the annotated pathology image decks as a learning tool” was included in the course surveys for the same cohort after the Fall blocks of their second year of medical school, including endocrine, renal, reproductive health, gastrointestinal, and liver blocks. Again, because of the limited number of free-text responses to survey questions, a descriptive thematic review was performed.

## Results

### Pre-implementation needs assessment

Over three semesters, including all organ system blocks, 70 free-text responses were submitted in course evaluations, 11 of which (16%) provided feedback regarding pathology/histology, including one positive comment (“path lectures were great!”), and ten other constructive responses, detailed in Table 1. Most comments were related to requests for annotated images pointing out histologic findings and assisting with the approach to interpreting abnormal histology (7/10, 70%), and the three remaining highlighted a need for more normal comparison and clinical tie-ins for context.

**Table 1:** Medical student free-text course survey feedback guided material design.

Free-text survey feedback from learners	Summary and learning material design
“Would appreciate more clear slides for pathology in terms of pointing out relevant findings” “For pathology, [have] one place for slides and annotations in lecture slides” “The pathology lectures should have more text on the slide, to facilitate student learning.” “With all pathology images, it would be so helpful to have more annotations of images.” “… Showing 1–2 slides and explaining exactly what we're looking for would be much more helpful.” "There needs to be a clear and methodical approach for teaching us how to read histology slides.” “Start with how to look at pathology/provide tips or a ‘cheat sheet’. There just didn't seem to be any systematic teaching of how to understand pathology specimens/slides.”	Annotated images pointing out histologic findings and providing a framework for understanding pathology images
“Require all pathology slides to have a reference to normal beside different pathology.” “This semester, most path slides were just an example of the disease, but it was missing the context on how we could have diagnosed/recognized the disease if we encountered this picture in the clinic or on an exam.” “The pathology (histology) lectures need to have better organization. It is not clear what we need to know and there is no framework or structure to put that information onto.”	More emphasis in pathology materials on normal histology for comparison with tie-in to clinical course content

#### ***Educational resource development***

##### **Slide deck creation**

A total of 511 annotated PowerPoint slides were created for ten distinct systems-based modules, covering 70 disease entities and ensuring tight integration of the pathology content with the rest of the curriculum (Supplemental Table 1). Systems-based modules included gastrointestinal, liver, reproductive, pulmonary, cardiovascular, neurology, endocrine, renal, skin, and musculoskeletal. The median number of slides per PowerPoint was 47, with a range of 21 (liver) to 78 (pulmonary). In total, 25 normal gross, 64 abnormal gross, 55 normal microscopic, and 203 abnormal microscopic images were included. Microscopic images included hematoxylin and eosin–stained and immunohistochemically stained sections, and the renal deck also included immunofluorescence and electron microscopy.

##### **Post-implementation learner evaluation**

Detailed survey results from the Spring and Fall quarters of 2022 are presented in Table 2. Respondents in the Spring 2022 cohort had completed pulmonary, cardiovascular, and musculoskeletal blocks. In this cohort, 50 of 77 students (65%) reported using the slide decks. Of those who used the resource, 32/50 (64%) either strongly agreed or agreed, 15/50 (30%) felt neutral, and 3/50 (6%) disagreed with the statement that the pathology decks were useful for learning (Table 2). During the second year of implementation, after completion of the endocrine, renal, reproductive health, gastrointestinal, and liver blocks, more students used the resource (66/85, 78%), and most (86%) thought they had good, very good, or excellent utility for learning. Of students who did use the resource, 50/70 (71%) agreed or strongly agreed that it was at the appropriate level for their learning.

**Table 2:** Learner feedback on slide decks: Structured responses.

Spring 2022: Pulmonary, cardiovascular and musculoskeletal block (77 respondents)								
The annotated pathology image slide decks available on Canvas were useful for learning.	I did not engage with this course element	Strongly disagree	Disagree	Neutral	Agree	Strongly agree	Likert mean (SD^a^)	No. of students who engaged in resource (%)
	27	0	3	15	18	14	3.86 (0.9)	50 (65%)

Fall 2022: endocrine, renal, reproductive health, gastrointestinal, and liver blocks (85 respondents)								

Please rate the utility of supplementary pathology PowerPoint PDFs (i.e., “endocrine pathology images”/" renal pathology images") provided by pathologists.	I did not engage with this course element	Poor	Fair	Good	Very good	Excellent	Likert mean (SD^a^)	No. of students who engaged in resource (%)

	19	5	4	18	20	19	3.67 (1.2)	66 (78%)

The annotated pathology PowerPoint slides were appropriate for my learning level for histopathology.	I did not engage with this course element	Strongly disagree	Disagree	Neutral	Agree	Strongly agree	Likert mean (SD^a^)	No. of students who engaged in resource (%)

	15	0	4	16	26	24	4 (0.9)	70 (82%)

a SD = standard deviation.

Table 3 summarizes positive and constructive free-text responses to the question “Please provide feedback for the Pathology Department about the annotated pathology image decks as a learning tool” issued to the Fall 2022 cohort. The positive feedback was related to overall enjoyment of the material and finding the annotations useful, making pathology material less intimidating and helpful for examinations. Students also shared that modules helped them integrate knowledge during examination preparation. The majority of the constructive to negative feedback related to wanting additional material and more detailed annotations, although two responses stated that the material was too extensive or that pathology was too heavily emphasized in their curriculum, and one respondent requested more guided exposure.

**Table 3:** Learning feedback on slide deck.

Summary of statements	Free-text responses
**Positive free-text responses:**	
Appreciation of annotations, comparison normals, and acceptability of the material	“The additional pathology slide decks are ∗amazing∗. They are SO clear … " “I particularly appreciated that there were notes on the side along with arrows directing my attention toward the pathological features of the image.” "I love that samples of healthy and pathological tissue are provided and labeled so that students can practice comparing." "The color coding is awesome. Thank you for taking the time to annotate." "Images [have] clear labels and key buzzwords for each disease, which is done really nicely in these slide decks.” “Having both gross and microscopic images is useful." “The decks are definitely good supplements with more clearly labeled and annotated images … "
Pathology material less intimidating	“Pathology is notoriously feared by medical students, so these extra resources are very much valued. Thanks!” “Very helpful as path is always my weakest link, thank you!”
Material helpful for integrating material	“… after I had reviewed the diseases, having a resource that took me step-by-step through the changes in pathological images was very rewarding.” “Very useful for exams” “These are very helpful for learning the key takeaways for each disease”
**Constructive free-text responses:**	
Interest in more detailed annotations	“I wish they were labeled even more. The more labels, the better. And the more times we could have an example of normal for comparison, the better!” “I would love more annotations on the images. I Found it a bit hard sometimes to know what parts of the image people were referring to.” “The path decks would be far more helpful if they were more thoroughly annotated … "
Interest in more repetition and versions of images	“Pathology is very challenging to me. I think, in general, repetition is key … Here's my recommendation: 1) KEEP the annotated slide deck exactly as is ….as a core slide deck with 1 slide representing each phenomenon 2) START a supplementary slide deck with other examples of the same phenomena for more repetition.” “The materials are really helpful to prepare for the labs and I felt very well guided. However, some images in the exams were so different that it was really hard to identify what it was.”
Too much detail and emphasis on pathology	“Way way way way too much pathology images, we do not need to know all of this path. We should be using that time to learn more about diseases and physiology. This is not a good use of time.” “Not clear why we do so much pathology. We are consistently told that we won't need to know X unless we become pathologists … Then we have to know it for tests. It takes so much more time to memorize pathology slides. I (and peers I've spoken to) spend an unbalanced amount of time trying to study pathology topics compared to the proportion of the course they are supposed to represent.”
In-person teaching better than independent learning	“Would honestly appreciate more guided exposure (i.e. someone who is actually telling you how to identify things) more frequently.”

## Discussion

Preclinical medical student learner feedback guided the creation of supplemental slide decks, including highly annotated microscopic and macroscopic visual images appropriate for early learners of histopathology. Our effort provides an initial learner perception assessment as an early indicator of curricular acceptability and feasibility. A majority of medical students used the resources, which included an increase of 13% from the first to the second quarter they were available. Feedback was generally positive, with most students reporting that the material was useful for their learning, had high utility, and was at the appropriate level for medical students. It specifically addresses the need for annotations, normal comparisons, and integration within a curriculum that incorporates pathology as a “thread” rather than as a stand-alone course.

Teaching an image-intensive discipline with reduced contact hours in the preclinical setting has been identified as a challenge in the literature.^21^, ^22^, ^23^ Students in our free-text responses emphasized appreciation of annotations and normal comparisons to increase their ability to understand and absorb high-yield content. An education team at a medical school in Jordan showed increased understanding, enjoyment, and engagement in practical histology classes with the association of side-by-side normals in their histology curriculum using PowerPoint.^21^ In our current curriculum, normal histology or “microscopic anatomy” is taught separately in a shorter course at the start of medical school, and histopathology is threaded into the three following quarters before the start of the clinical years. Our students require review of their normal histology material to effectively learn the abnormal histology in our curriculum. In addition to our teaching slide decks, we have required all pathology didactic material to include side-by-side normal comparisons for gross and microscopic images wherever possible.

A growing body of literature supports the use of digital resources to enhance pathology and histology education, including virtual microscopy, interactive web-based platforms, adaptive tutorials, collaborative annotation tools, and asynchronous e-learning modules.^14^^,^^17^^,^^18^^,^^24^, ^25^, ^26^ Studies cite the boon of digital pathology in pathology medical education, demonstrating improved student understanding and performance and describing positive teacher and student perceptions relative to light microscopy.^27^^,^^28^ However, many existing digital pathology resources are designed as stand-alone learning tools, virtual microscopy platforms with whole-slide images, or board preparation resources rather than as materials integrated into a specific preclerkship organ systems curriculum. For example, several excellent resources use whole-slide images for self-guided learning for medical students and pathology trainees.^6^^,^^17^^,^^18^^,^^29^, ^30^, ^31^ Additionally, several excellent resources targeted to advanced medical students or postgraduate pathology trainees have been developed.^9^^,^^24^^,^^25^^,^^32^ Despite the existence of these resources, survey results from medical students at our institution expressed feeling overwhelmed by the pathology curriculum, stating a need for basic skills in “how to look at pathology” and “systematic teaching of how to understand pathology specimens/slides” (Table 1).

Our educational intervention complements these existing resources by addressing a different educational need. Rather than replacing lectures or laboratory sessions, the annotated slide decks were developed in direct response to learner feedback and designed to reinforce concurrent organ system teaching within our curriculum. Emphasis was placed on side-by-side comparison of normal and abnormal findings, detailed image annotations highlighting key diagnostic features, and concise clinical correlations appropriate for undergraduate medical learners. This resource was paired with small-group laboratory sessions to provide an associated active learning component. In the small groups, which were each facilitated by one clinician and one pathologist, case-based questions and prompts regarding the clinical workup and pathologic findings were presented to students for facilitated discussion. After the small-group laboratories, the case-based questions and pathology slide decks remained available for later individual study.

The importance of preclinical pathology education as imprinting exposure to our field among many medical students is recognized. Therefore, our project aimed to make pathology more accessible, simple, straightforward, and enjoyable for the students, replete with many colors and clear tie-ins to their organ system–based curriculum. One way we did this was to carefully curate the list of entities covered to provide an iterative review of concepts also covered in the didactic and small-group case-based sessions, with alignment between the pathology thread lead and organ system–based block director leads.

This brings us to a limitation of our effort, which is adaptability and generalizability. Creating the resource required many hours of work over months by a pathology team dedicated to education. Finding ideal images and normal comparisons and adding annotations is time intensive. Our team included senior residents and pathology fellows overseen by pathology faculty leads in the preclinical curriculum. In the future, our team aims to collaborate with the computational pathology and digital innovations team in our department in the hope that it will streamline the curation and annotation of teaching materials and reduce the time involved in these efforts.^33^ Although the slide decks were developed for our institution's curriculum, we believe the underlying educational design process could be broadly reproducible. Educators may adapt the underlying design principles to their own curricula by identifying learner needs through curriculum evaluation, selecting representative normal and pathologic images from either institutional or publicly available images, incorporating concise image annotations and clinical correlations from academic pathologists, and integrating the materials alongside existing lectures and, particularly, small-group activities to promote active learning. Incorporating small-group discussion and/or interactive questions that require learners to actively identify and interpret pathologic findings, rather than passively review annotated images, is an important consideration for improving the educational value of this resource.

Another key limitation of this study is that our evaluation focused on learner perceptions and acceptability rather than objective measures of educational effectiveness. The survey questions were incorporated into the institution's existing required end-of-course evaluations, which limited the scope and number of pathology-specific questions that could be included. Our primary aim was to determine whether the educational resource addressed the needs identified during the initial curriculum review and whether students perceived it as useful and appropriate for their level of training. While positive learner perceptions are an important indicator of curricular feasibility and acceptability, they do not establish improved learning outcomes or knowledge retention. Future studies evaluating objective educational outcomes such as examination performance, image interpretation skills, and knowledge retention would provide additional value. However, such studies may be challenging to conduct within a required integrated curriculum. Alternative study designs, such as comparisons across successive cohorts or evaluation of performance before and after implementation, may provide additional insight into educational effectiveness. While we attempted to evaluate Step 1 scores across the pre- and post-implementation cohorts, this information is not publicly available, and the USMLE score change in January 2022 from a 3-digit score to a pass/no-pass scoring system represents a significant confounding variable.

In future efforts, we hope to improve our materials by adding, as the learners suggested in their feedback, more image examples and even more annotations. We also hope to increase the clinical relevance and “warmth factor” of the content by adding more clinical tie-ins and having the content be delivered in person by our most enthusiastic pathology educators in didactic and case-based sessions to facilitate personal mentorship. As one student stated, they “would honestly appreciate more guided exposure (i.e., someone who is actually telling you how to identify things) more frequently.” Additionally, incorporating image-based formative assessment questions with immediate feedback could further enhance learner engagement and educational impact. Our next step is to collaborate with the Department of Radiology to expand upon their efforts to extract digital radiology and pathology images from patient cases contained within our electronic medical records and imaging systems.^34^ This effort aims to enhance medical student learning and facilitate the development of curriculum content. By integrating these images into clinical case contexts, we aim to enrich the educational experience, providing a robust platform for both formative and summative assessment of the material covered.

A preclinical medical student's path to pathology includes pursuing shadowing opportunities in pathology after a particularly riveting didactic session led by a pathologist. Curating material that highlights the integral relevance of our field and delivered by a pathologist who shares clinical stories can pave the way for shadowing opportunities and eventually the experiential learning that has been shown to be the key to recruitment.^35^ By tailoring our pathology visual teaching materials to the needs of preclinical students and simplifying the learning process, we can make our field more accessible, perhaps one PowerPoint slide at a time. Correlating our preclinical pathology material developments with examination performance, perception survey data, and eventually residency selection data will be helpful to measure the impact of innovations.

## Conclusions

Our teaching intervention highlights ongoing challenges regarding pathology education in undergraduate medical education and provides practical solutions. Survey data conveyed generally positive student feedback regarding the supplemental pathology image decks, with utilization by most students, underscoring the value of the modules in addressing the originally identified pathology resource gap. Students also provided constructive insights into areas where the current material could be further enhanced, including better advertising of the resources. Ongoing collaboration between specialty clinicians and pathology educators using institutional digital gross and histology image archives will enhance visual pathology learning resources for preclerkship medical students.

## Ethics declaration

Written informed consent to take part in the study and to publish the article has been obtained from all participants or their legal representatives. The privacy rights of participants have been observed.

This study was performed in compliance with relevant laws, regulatory frameworks, and guidelines where the research took place. The Stanford institutional review board (IRB) granted an exemption. The authors provided the following explanation: Per Stanford IRB protocol: Medical education projects using anonymous/confidential surveys and evaluations fall under exempt category after medical education leadership group review, which was obtained. Our IRB number is recorded in the article (protocol number 78315).

## Statement regarding the use of artificial intelligence

No artificial intelligence (AI) tools or generative AI technologies were used in the preparation or writing of this article.

## Funding

The article processing fee for this article was funded by an Open Access Award given by the Society of ‘67, which supports the mission of the Association for Academic Pathology to produce the next generation of outstanding investigators and educational scholars in the field of pathology. This award helps to promote the publication of high-quality original scholarship in *Academic Pathology* by authors at an early stage of academic development.

## Declaration of competing interests

The authors declare that they have no known competing financial interests or personal relationships that could have appeared to influence the work reported in this paper.
