# Supplementary material for: A picture is worth 1000 pathological learning points: Creating visual pathology teaching materials appropriate for preclerkship medical student learning
Source: Acad Pathol. 2026 Oct 17;13(4):100305. doi: 10.1016/j.acpath.2026.100305 (PMC13696811; doi:10.1016/j.acpath.2026.100305)
Supplement: Supplementary file 1 [file mmc1.docx]

| **Supplemental Table 1. Slide deck visual content summary.** | |
| --- | --- |
| **Block** | **Entities covered** |
| Cardiovascular | Normal heart Myocardial infarction - early Myocardial infarction - late Coronary artery atherosclerosis Ventricular hypertrophy Dilated cardiomyopathy Cardiac amyloidosis Viral myocarditis |
| Pulmonary | Normal lung Pulmonary edema & ARDS Pulmonary infarct Emphysema Chronic obstructive pulmonary disease Usual interstitial pneumonia Asbestosis Tuberculosis Bronchopneumonia Lobar pneumonia Aspergillosis Adenocarcinoma of lung Squamous carcinoma of lung Small cell carcinoma of lung Mesothelioma |
| Musculoskeletal | Normal bone and muscle Rhabdomyoma Rhabdomyosarcoma Leiomyoma Leiomyosarcoma Lipoma Liposarcoma Osteosarcoma Chondrosarcoma Undifferentiated sarcoma Ewing sarcoma Hemangioma Angiosarcoma Rheumatoid arthritis Duchenne muscular dystrophy |
| Endocrine | Thyroid, normal Chronic lymphocytic thyroiditis Benign thyroid nodule (adenoma) Papillary thyroid carcinoma Follicular carcinoma Medullary carcinoma Anaplastic thyroid carcinoma Parathyroid gland Parathyroid adenoma Adrenal, normal Pheochromocytoma Adrenal cortical adenoma Normal pancreas Pancreatic neuroendocrine tumor Pituitary Pituitary adenoma Craniopharyngioma |
| Gastrointestinal | Esophagus Gastrointestinal reflux disease Esophageal squamous cell carcinoma Esophageal adenocarcinoma Stomach H. pylori gastritis Autoimmune gastritis Atrophic gastritis Peptic ulcer disease Gastric cancer Large intestine normal Small intestine normal Celiac disease Colon, normal Crohn disease Ulcerative colitis Colorectal adenocarcinoma Pancreas, normal Acute/chronic pancreatitis Liver: Normal liver Cirrhosis Chronic hepatitis B Alcoholic cirrhosis Drug induced liver injury |
| Neurology | Alzheimer disease Parkinson disease Huntington disease Multiple sclerosis Ischemic brain injury Medulloblastoma Glioblastoma Oligodendroglioma Pituitary adenoma (from endocrine) Craniopharyngioma (from endocrine) |
| Renal | Normal kidney Acute tubular injury Minimal change disease Diabetic nephropathy Focal segmental glomerulosclerosis Membranous nephropathy Amyloidosis IgA nephropathy Infection-associated glomerulonephritis (acute poststreptococcal glomerulonephritis) Rapidly progressive glomerulonephritis (ANCA vasculitis, anti-GBM disease, Lupus nephritis) Membranoproliferative glomerulonephritis (mixed nephrotic-nephritic) Alport syndrome |
| Reproductive | Normal ovary Functional ovarian cyst Normal secretory & proliferative endometrium Endometrial hyperplasia Endometrioid carcinoma Endometriosis Normal uterine wall Leiomyoma Normal fallopian tube Ectopic pregnancy Normal cervix LSIL HSIL Invasive squamous cell carcinoma Normal placenta Molar pregnancy Normal prostate Prostate cancer Normal breast Ductal carcinoma in situ Invasive ductal carcinoma |
| Skin | Normal skin Intradermal nevus Invasive melanoma Basal cell carcinoma Acrochordon Angioma Seborrheic keratosis Psoriasis Connective tissue disease (lupus and dermatomyositis) |
