# Supplementary material for: A picture is worth 1000 pathological learning points: Creating visual pathology teaching materials appropriate for preclerkship medical student learning
Source: Acad Pathol. 2026 Oct 17;13(4):100305. doi: 10.1016/j.acpath.2026.100305 (PMC13696811; doi:10.1016/j.acpath.2026.100305)
Supplement: Supplementary file 2 [file mmc2.docx]

**Supplemental Material 1**

End of Course Survey Questions Related to Pathology (before pathology resources)

Science of Medicine Courses: 2020-2021

**Please provide comments on what course directors should “keep-stop-start” and why.**

Qualitative, free-text response

End of Course Survey Questions Related to Pathology (after pathology resources)

Science of Medicine Course: 2021-2022

**Please rate the following course elements:**

**The annotated pathology image slide decks available on Canvas were useful for learning**

Answer choices: N/A-did not use, strongly disagree, disagree, neutral, agree, strongly agree

End of Course Survey Questions Related to Pathology (after pathology resources)

Science of Medicine Courses: 2022-2023

**This year, the pathology department prepared a slide deck for each block with**

**pathology images that are labeled/annotated. These PDF slide decks are under**

**"Pathology Resources" on Canvas. We greatly appreciate your feedback on this effort.**

**Please rate the utility of supplementary pathology PowerPoints PDFs i.e., "Endocrine Pathology Images" / " Renal Pathology Images" provided by Pathologists**

Answer choices: I did not engage with this course element, poor, fair, good, very good, excellent

**The annotated pathology PowerPoint slides were appropriate for my learning level for histopathology.**

Answer choices: I did not engage with this course element, strongly disagree, disagree, neutral, agree, strongly agree

**Please provide feedback for the Pathology Department about the annotated pathology image decks as a learning tool.**

Qualitative, free-text response
